# Primary Bladder Lymphoma with Extravesical Extension: A Case Report and Literature Review on Prognosis and Clinical Characteristics

**DOI:** 10.3390/jcm13154340

**Published:** 2024-07-25

**Authors:** Hideshige Seki, Shohei Mizuno, Sakura Saigusa, Yukie Sugita, Yusuke Iida, Saki Shinohara, Kaori Uchino, Tomohiro Horio, Ichiro Hanamura, Akiyoshi Takami

**Affiliations:** Division of Hematology, Department of Internal Medicine, Aichi Medical University, Nagakute 480-1195, Japan; seki.hideshige.083@mail.aichi-med-u.ac.jp (H.S.); kozaki.sakura.269@mail.aichi-med-u.ac.jp (S.S.); yamada.yukie.913@mail.aichi-med-u.ac.jp (Y.S.); iida.yuusuke.240@mail.aichi-med-u.ac.jp (Y.I.); yamada.saki.097@mail.aichi-med-u.ac.jp (S.S.); ksakai@aichi-med-u.ac.jp (K.U.); kuroro@aichi-med-u.ac.jp (T.H.); hanamura@aichi-med-u.ac.jp (I.H.); takami-knz@umin.ac.jp (A.T.)

**Keywords:** primary bladder lymphoma, diffuse large B-cell lymphoma, extravesical extension, prognosis, urinary tract infection, case report, literature review

## Abstract

**Background:** Primary bladder lymphoma is generally regarded as having a favorable prognosis due to the predominance of low-grade lymphomas confined to the bladder. However, our investigation reveals that cases with extravesical extension, predominantly involving diffuse large B-cell lymphoma (DLBCL), exhibit a distinct clinical course with varied prognostic outcomes. **Methods:** In this report, we present and analyzed the clinical features and outcomes of 47 patients with primary bladder lymphoma with extravesical extension, including the case that we experienced. **Results:** An 77-year-old man who experienced fever, anorexia, and general malaise was referred to our hospital. Initial laboratory tests indicated severe renal failure, pyuria, and *Escherichia coli* bacteremia, accompanied by diffuse thickening of the bladder walls and increased attenuation in the surrounding adipose tissues. Initially misdiagnosed with a severe urinary tract infection leading to sepsis, the patient was treated with antibiotics and hemodialysis. Upon readmission due to abdominal pressure, imaging identified an intra-abdominal mass connected to the bladder wall. A bladder biopsy was performed, resulting in the diagnosis of primary bladder DLBCL with perivesical extension, classified as germinal center B-cell type. Taking inspiration from this case, the review of 46 patients was implemented. As a result, we resolved that primary bladder lymphoma often includes indolent types like Mucosa-associated lymphoid tissue lymphoma, but cases with extravesical expansion are predominantly DLBCL. **Conclusions:** This case emphasizes the diagnostic complexities of distinguishing primary bladder lymphoma from urinary tract infections and underscores the prognostic implications of extravesical extension. Our comprehensive review of the literature on primary bladder lymphomas with extravesical involvement highlights the clinical characteristics, therapeutic challenges, and need for heightened diagnostic vigilance and tailored treatment strategies for this subset of patients.

## 1. Introduction

Diffuse large B-cell lymphoma (DLBCL) is subdivided within the International Consensus Classification 2022, World Health Organization fourth and beta fifth edition [1,2,3]. These categories are decided by various examinations and laboratory tests, such as histopathological findings, genetic mutations, and the primary lesion of the disease. However, lymphoma of the bladder is not stated in detail by these classifications. Primary bladder lymphoma constitutes only 0.2% of all lymphomas and exhibits a distinct female-to-male ratio of 3:1, contrasting with the overall lymphoma ratio of 1:1.3 that favors men [4,5,6]. This rare condition primarily manifests symptoms commonly associated with urinary tract infections. Mucosa-associated lymphoid tissue (MALT) lymphoma/marginal zone lymphoma (MZL) accounts for over 80% of primary bladder lymphoma cases, is generally localized within the bladder cavity, and is associated with a favorable prognosis. However, primary bladder lymphoma with perivesical expansion complicates diagnosis and can lead to rapid disease progression, often necessitating chemotherapy [7]. Comprehensive reports on primary bladder lymphoma with perivesical expansion are scarce, and its clinical pathology is not well understood. We report a case of primary bladder lymphoma with perivesical expansion that presented with clinical symptoms and imaging findings akin to those of a urinary tract infection; we also include a review of the literature.

## 2. Case Presentation

**Figure 1:**
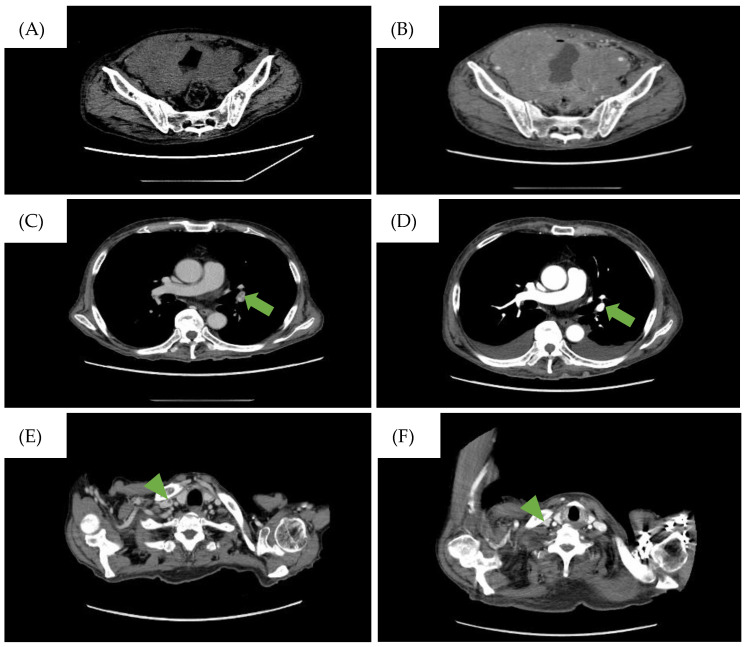
CT findings during the clinical course of the patient. (**A**) CT scan on the day the patient was referred to our hospital. Diffuse bladder wall thickening up to 7 mm was observed. (**B**) CT scan taken when the patient complained of abdominal pressure. A large intra-abdominal mass was observed. The low-density area represents the pulmonary embolism (PE) in the (**C**) left pulmonary artery and (**E**) the right internal carotid artery and disappearance of the PE after treatment in the (**D**) left pulmonary artery (→) and (**F**) the right internal carotid artery (▶︎).

A 77-year-old man was referred to our hospital with fever, anorexia, and general malaise. Initial laboratory tests indicated severe renal failure, elevated levels of C-reactive protein and soluble interleukin-2 receptor, pyuria, and bacteremia due to *Escherichia coli* (Table 1). No malignant cells were confirmed from the urine cytology test. Computed tomography (CT) scans showed thickening of the bladder wall and increased opacity surrounding the bladder (Figure 1A), and pathological conditions such as trabeculation, malignant tumors, and infections including cystitis were cited as a differential diagnosis. However, he was diagnosed with sepsis associated with a severe urinary tract infection due to the laboratory findings mentioned above. Treatment for an infection by antibiotics and hemodialysis for uremia were carried out. His renal function and general condition improved after these treatments, leading to his discharge one month after admission.

**Figure 2:**
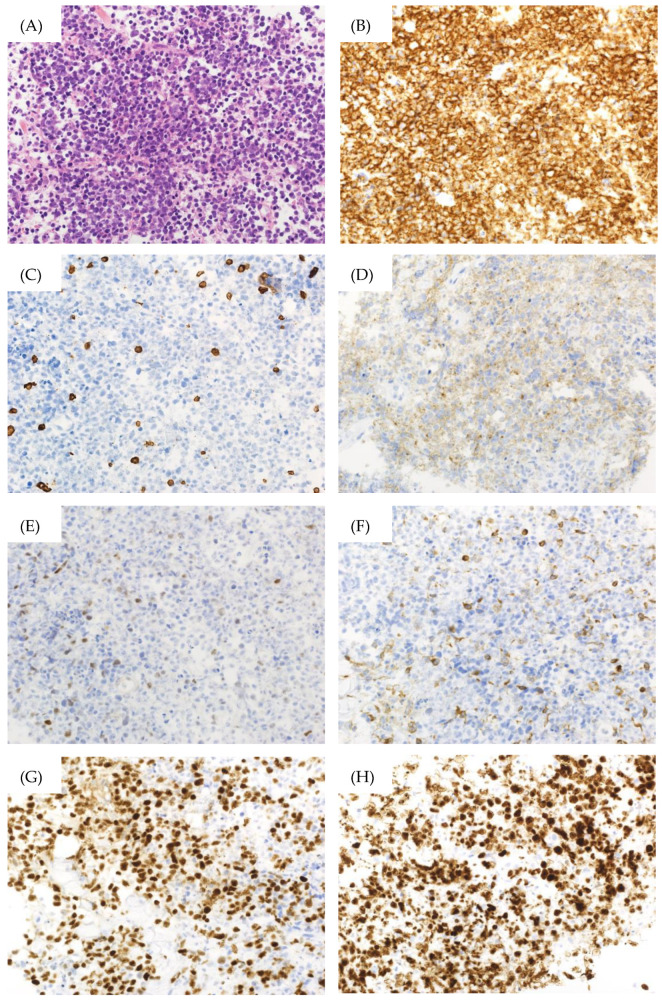
Pathological findings of the bladder biopsy specimen. (**A**) Hematoxylin–Eosin Stain (×400), (**B**) CD20 immunostaining (×400), (**C**) CD3 immunostaining (×400), (**D**) CD10 immunostaining (×400), (**E**) c-MYC immunostaining (×400), (**F**) BCL2 immunostaining (×400), (**G**) BCL6 immunostaining (×400), (**H**) Ki-67 immunostaining (×400).

However, the patient returned a week later, reporting abdominal pressure. Follow-up CT scans revealed a large intra-abdominal mass attached to the bladder wall (Figure 1B), and deep venous thrombosis (DVT) in the bilateral lower extremities, pulmonary embolism (PE) in the left pulmonary artery, and arterial thrombosis of the right internal carotid artery were also identified (Figure 1C). These thromboses were likely due to compression of the bilateral internal iliac veins by the tumor and tumor-induced coagulation abnormalities. A CT-guided biopsy of the mass confirmed this, and pathological findings showed diffuse infiltration of large, atypical cells with CD20-, CD10-, and BCL6-positive and CD3-, c-MYC-, BCL2-negative immunostaining. The Ki-67 index was 90% in these specimens (Figure 2). Therefore, the diagnosis of DLBCL was made. Further imaging studies showed no lymphadenopathy, and bone marrow infiltration tests were negative, establishing a diagnosis of primary bladder DLBCL, stage IE (bulky) according to the Ann Arbor classification [8], a favorable Revised International Prognostic Index (calculated by age, lactate dehydrogenase levels, performance status, Ann Arbor staging, and number of extra nodal lesions; evaluated as 0 points: good, 1–2 points: favorable, 3 points or more: poor) due to age and high lactate dehydrogenase levels, and germinal center B-cell subtype (CD10-positive).

Treatment with R-CHOP regimen (rituximab, cyclophosphamide, doxorubicin, vincristine, and prednisone) was initiated alongside warfarin and heparinization due to thrombosis risk. After one cycle of R-CHOP, a partial response was observed, and the PE resolved (Figure 1D). However, after three cycles (three months after cycle one started), the patient experienced severe desaturation and a massive pleural effusion was detected on a CT scan. Large lymphocytes with constriction in the nucleus and a distinct nucleolus were found in the thoracentesis sample and confirmed a DLBCL relapse. Given the patient’s limited physical condition and preferences, invasive treatments were halted in favor of best supportive care.

## 3. Discussion

The patient was initially diagnosed with a severe urinary tract infection based on clinical and imaging findings. However, subsequent evaluations revealed a primary bladder lymphoma of the DLBCL type with perivesical expansion. Additionally, DVT in both lower extremities and PE were observed. The enlargement of the bladder lymphoma caused narrowing of the internal iliac veins on the dorsal side of the bladder, which likely contributed to the development of DVT. To our knowledge, this is the first reported case of primary bladder lymphoma complicated by venous thromboembolism. Lymphoma is a known risk factor for arteriovenous thrombosis [9,10,11], as evidenced by thrombosis in the bilateral femoral veins and internal carotid vein in this patient. This case indicates that the incidence of lower-extremity DVT due to direct compression of the internal iliac veins by bladder lymphoma, as shown in the current case, may be underestimated.

**Figure 3:**
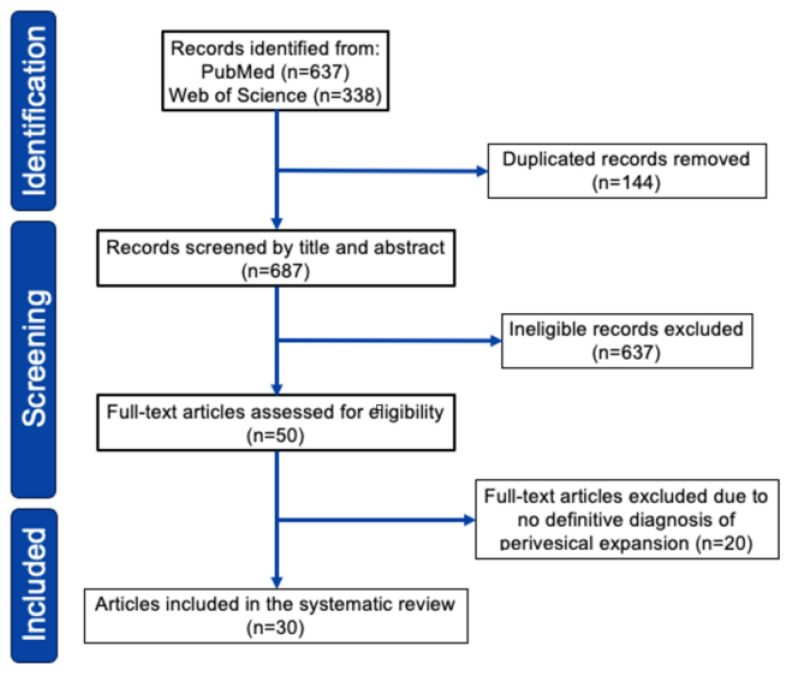
PRISMA 2020 flowchart of study selection for the systematic review.

Our search identified 750 relevant citations which were subsequently screened, resulting in 74 articles selected for full-text review. After full-text review, we identified 30 pertinent studies in which data extraction was possible, published between 1997 and 2024 (Figure 3, see Supplementary Document S1 and Table S2 for detail). As shown in Table 2 (see Supplementary Table S1 for detail), we reviewed 47 cases of primary bladder lymphoma with perivesical expansion, including this case [5,6,7,12,13,14,15,16,17,18,19,20,21,22,23,24,25,26,27,28,29,30,31,32,33]. The female-to-male ratio was 2.2:1, and the median age was 66 years (range 27–89 years). Initial presentations included hematuria (52%), anuria/oliguria/hydronephrosis (19%), urinary tract infection (10%), urinary urgency (4%), and low back/abdominal pain (15%). Urinary tract infection (UTI)-related symptoms, including hematuria and urinary urgency, were present in 60% of patients. The most common histological type was DLBCL (43%), followed by MALT lymphoma/MZL (36%). Regarding treatment, 30 of 47 patients (64%) received chemotherapy, and 15 patients (31%) received radiotherapy. Of the 30 patients who received chemotherapy, 22 (73%) responded. Of the 43 patients treated, 32 (74%) survived, and 10 (23%) died or showed progressive disease within 6–20 months. Regarding the relationship between histological type and survival rate, 14 of 20 DLBCL cases (70%) survived, and 13 of 17 MZL cases (76%) survived. These outcomes were considered comparable to the typical chemotherapy results for DLBCL and MZL.

Therefore, if a patient has recurrent UTIs, severe infections as described here, or general malaise and abdominal symptoms disproportionate to a UTI, the possibility of primary bladder lymphoma with perivesical expansion should be considered. Additionally, diagnosing this type of lymphoma can be difficult in the presence of perivesical extension but no intravesical involvement, as cystoscopy and superficial lymph node biopsy may be insufficient. In these cases, more invasive procedures such as deep biopsy or open biopsy may be necessary. An important limitation of this report is that the patient’s clinical course progressed rapidly after discharge, which did not allow sufficient time for metabolic imaging scans, such as positron emission tomography (PET) scans. PET scans may help assess the need for biopsy of extravesical tumors and determine the urgency of such a procedure. Primary bladder lymphoma with perivesical expansion often presents with symptoms similar to those of a urinary tract infection, such as fever, hematuria, pain during urination, and frequent urination, and these symptoms are commonly observed in cystitis cases as well. Unlike primary bladder lymphoma, which remains confined within the bladder cavity and progresses slowly with a good survival rate, primary bladder lymphoma with perivesical expansion typically manifests as an intermediate-grade histological type, such as DLBCL, and tends to progress rapidly, often necessitating chemotherapy, as demonstrated in this report. If a patient experiences repeated urinary tract infections, severe infections like those documented here, or general malaise and abdominal symptoms disproportionate to urinary infections, it is essential to consider the possibility of primary bladder lymphoma with perivesical expansion. Moreover, diagnosing this type of lymphoma can be challenging in cases with perivesical expansion but no intravesical lesions, as cystoscopy or superficial lymph node biopsy may be insufficient. In such cases, more invasive procedures such as deep biopsy or open biopsy may be necessary.

In conclusion, primary bladder lymphoma with extravesical expansion is difficult to differentiate from UTIs, particularly in early-stage cases like the one presented here. While primary bladder lymphoma often includes indolent types like MALT lymphoma, cases with extravesical expansion are predominantly DLBCL. This study provides a comprehensive understanding of primary bladder lymphoma with perivesical expansion and emphasizes the need for careful differential diagnosis to avoid misdiagnosis and ensure appropriate treatment.

## Figures and Tables

**Table 1:** Blood test results at the time of hospitalization.

Complete Blood Count		
White blood cell	4300/μL	3300–8600/μL
Red blood cell	2.62 × 10^6^/μL	4.35–5.55 × 10^6^/μL
Hemoglobin	8.9 g/dL	13.7–16.8 g/dL
Hematocrit	28.70%	40.7–50.1%
Mean corpuscular volume	109.5 fL	83.6–98.2 fL
Mean corpuscular hemoglobin concentration	31.0%	31.7–35.3%
Platelet	180 × 10^3^/μL	158–348 × 10^3^/μL
Coagulation		
Prothrombin time	12.1 s	10–13 s
Prothrombin time international ratio	0.95	0.85–1.15
Activated partial thromboplastin time	34.0 s	22.4–39.2 s
Fibrinogen	145 mg/dL	190–330 mg/dL
Seroimmunological test		
C-reactive protein	4.48 mg/dL	0.00–0.14 mg/dL
Biochemistry		
Total protein	6.2 g/dL	6.6–8.1 g/dL
Albumin	2.8 g/dL	4.1–5.1 g/dL
Total bilirubin	0.54 mg/dL	0.40–1.50 mg/dL
Urea nitrogen	28.3 mg/dL	8.0–20.0 mg/dL
Uric acid	5.1 mg/dL	3.7–7.0 mg/dL
Creatinine	5.26 mg/dL	0.65–1.07 mg/dL
Estimated glomerular filtration rate	9 mL/min/1.73^2^	>90 mL/min/1.73^2^
Sodium	138 mmol/L	138–145 mmol/L
Potassium	4.2 mmol/L	3.6–4.8 mmol/L
Chloride	102 mg/dL	101–108 mmol/L
Aspartate aminotransferase	41 U/L	13–30 U/L
Alanine aminotransferase	6 U/L	10–42 U/L
Lactate dehydrogenase	608 U/L	124–222 U/L
γ-glutamyl transpeptidase	12 U/L	13–64 U/L
Creatinine kinase	15 U/L	59–248 U/L
Soluble interleukin-2 receptor	14,617 U/mL	122–496 U/mL

**Table 2:** Characteristics of patients with primary bladder lymphoma with perivesical expansion.

Age, median (range), years	66 (27–89)
Female-to-male ratio	2.2:1
Initial presentation, N (%)	
Hematuria	24 (52)
Anuria/Oliguria/Hydronephrosis	9 (19)
Urinary tract infection	4 (10)
Urinary urgency	2 (4)
Low back/abdominal pain	7 (15)
Histological type, N (%)	
DLBCL	20 (43)
MALT/MZL	17 (36)
Others	10 (21)
Treatment strategy	
Chemotherapy	30 (64)
Radiotherapy	15 (31)
Others	2 (4)
Treatment respondence	
>PD	22 (73)
<SD or NA	8 (17)

DLBCL; diffuse large B-cell lymphoma; MALT, mucosa-associated lymphoid tissue; MZL, marginal zone lymphoma; PD, progressive disease; SD, stable disease; NA, not available.

## Data Availability

The authors confirm that the data supporting the findings of this study are available within the article and its Supplementary Materials.

## Supplementary Materials

The following supporting information can be downloaded at: https://www.mdpi.com/article/10.3390/jcm13154340/s1, Document S1: Literature Review Method and Study Selection [34]; Table S1: Characteristics of patients with primary bladder lymphoma with perivesical expansion; Table S2: PRISMA statement.

## References

[1] Campo E., Jaffe E.S., Cook J.R., Quintanilla-Martinez L., Swerdlow S.H., Anderson K.C., Brousset P., Cerroni L., de Leval L., Dirnhofer S. (2022). The International Consensus Classification of Mature Lymphoid Neoplasms: A report from the Clinical Advisory Committee. Blood.

[2] Alaggio R., Amador C., Anagnostopoulos I., Attygalle A.D., Araujo I.B.O., Berti E., Bhagat G., Borges A.M., Boyer D., Calaminici M. (2022). The 5th edition of the World Health Organization Classification of Haematolymphoid Tumours: Lymphoid Neoplasms. Leukemia.

[3] Swerdlow S.H., Campo E., Pileri S.A., Harris N.L., Stein H., Siebert R., Advani R., Ghielmini M., Salles G.A., Zelenetz A.D. (2016). The 2016 revision of the World Health Organization classification of lymphoid neoplasms. Blood.

[4] Al-Maghrabi J., Kamel-Reid S., Jewett M., Gospodarowicz M., Wells W., Banerjee D. (2001). Primary low-grade B-cell lymphoma of mucosa-associated lymphoid tissue type arising in the urinary bladder: Report of 4 cases with molecular genetic analysis. Arch. Pathol. Lab. Med..

[5] Bates A.W., Norton A.J., Baithun S.I. (2000). Malignant lymphoma of the urinary bladder: A clinicopathological study of 11 cases. J. Clin. Pathol..

[6] Kempton C.L., Kurtin P.J., Inwards D.J., Wollan P., Bostwick D.G. (1997). Malignant lymphoma of the bladder: Evidence from 36 cases that low-grade lymphoma of the MALT-type is the most common primary bladder lymphoma. Am. J. Surg. Pathol..

[7] Hughes M., Morrison A., Jackson R. (2005). Primary bladder lymphoma: Management and outcome of 12 patients with a review of the literature. Leuk. Lymphoma.

[8] Armitage J.O. (2005). Staging non-Hodgkin lymphoma. CA Cancer J. Clin..

[9] Khorana A.A., Mackman N., Falanga A., Pabinger I., Noble S., Ageno W., Moik F., Lee A.Y.Y. (2022). Cancer-associated venous thromboembolism. Nat. Rev. Dis. Primers.

[10] Gervaso L., Dave H., Khorana A.A. (2021). Venous and Arterial Thromboembolism in Patients With Cancer: JACC: CardioOncology State-of-the-Art Review. JACC CardioOncol..

[11] Caruso V., Di Castelnuovo A., Meschengieser S., Lazzari M.A., de Gaetano G., Storti S., Iacoviello L., Donati M.B. (2010). Thrombotic complications in adult patients with lymphoma: A meta-analysis of 29 independent cohorts including 18 018 patients and 1149 events. Blood.

[12] Arda K., Ozdemir G., Güneş Z., Ozdemir H. (1997). Primary malignant lymphoma of the bladder. A case report and review of the literature. Int. Urol. Nephrol..

[13] Mourad W.A., Khalil S., Radwi A., Peracha A., Ezzat A. (1998). Primary T-cell lymphoma of the urinary bladder. Am. J. Surg. Pathol..

[14] Tasu J.P., Geffroy D., Rocher L., Eschwege P., Strohl D., Benoit G., Paradis V., Bléry M. (2000). Primary malignant lymphoma of the urinary bladder: Report of three cases and review of the literature. Eur. Radiol..

[15] Sönmezer M., Ensari A., Ustün Y., Güngör M., Ortaç F. (2002). Primary lymphoma of the urinary bladder presenting as a large pelvic mass. J. Pak. Med. Assoc..

[16] Choi J.H., Jeong Y.Y., Shin S.S., Lim H.S., Kang H.K. (2003). Primary calcified T-cell lymphoma of the urinary bladder: A case report. Korean J. Radiol..

[17] Oh K.C., Zang D.Y. (2003). Primary non-Hodgkin’s lymphoma of the bladder with bone marrow involvement. Korean J. Intern. Med..

[18] Leite K.R., Bruschini H., Camara-Lopes L.H. (2004). Primary lymphoma of the bladder. Int. Braz. J. Urol..

[19] Froehner M., Haase M., Hakenberg O.W., Wirth M.P. (2004). Urinary immunocytology for primary bladder B cell lymphoma. Urology.

[20] Riccioni R., Carulli G., de Maria M., Pacini S., Cagno C., Selli C., Petrini M. (2006). Primary lymphoma of the bladder: Case report. Am. J. Hematol..

[21] Evans D.A., Moore A.T. (2007). The first case of vesico-vaginal fistula in a patient with primary lymphoma of the bladder—A case report. J. Med. Case Rep..

[22] Terzic T., Radojevic S., Cemerikic-Martinovic V., Stevanovic R., Citlucanin S., Mitrovic D., Stojimirovic B., Markovic-Lipkovski J. (2008). Primary non-hodgkin lymphoma of urinary bladder with nine years later renal involvement and absence of systemic lymphoma: A case report. Med. Oncol..

[23] Horasanli K., Kadihasanoglu M., Aksakal O.T., Ozagari A., Miroglu C. (2008). A case of primary lymphoma of the bladder managed with multimodal therapy. Nat. Clin. Pract. Urol..

[24] Hayashi A., Miyakawa Y., Bokuda K., Kimura T., Nakashima E., Irie R., Sugiura H., Suzuki T., Ohsone Y., Akizuki S. (2009). Primary Diffuse Large B-Cell Lymphoma of the Bladder. Intern. Med..

[25] Díaz-Peromingo J.A., Tato-Rodríguez J., Pesqueira-Fontán P.M., Molinos-Castro S., Gayol-Fernández M.C., Struzik J.P. (2010). Non-Hodgkin’s lymphoma presenting as a primary bladder tumor: A case report. J. Med. Case Rep..

[26] Wang L., Cao Z.Z., Qi L. (2011). Primary T-cell lymphoma of the urinary bladder presenting with haematuria and hydroureteronephrosis. J. Int. Med. Res..

[27] Nerli R.B., Guntaka A.K., Das S., Hiremath M.B. (2013). Primary non-Hodgkin lymphoma of the bladder. Indian J. Cancer.

[28] Mahfoud T., Tanz R., Mesmoudi M., Khmamouche M.R., El Khannoussi B., Ichou M., Errihani H. (2013). Primary non-Hodgkin’s lymphoma of the bladder: Case report and literature review. Pan. Afr. Med. J..

[29] Simpson W.G., Lopez A., Babbar P., Payne L.F. (2015). Primary bladder lymphoma, diffuse large B-cell type: Case report and literature review of 26 cases. Urol. Ann..

[30] Ogihara K., Kosaka T., Kikuchi E., Hongo H., Mikami S., Oya M. (2016). Spontaneous Regression of Epstein-Barr Virus-Positive Primary Diffuse Large Cell B-Cell Lymphoma of the Urinary Bladder After the Cessation of Enzalutamide. Clin. Genitourin. Cancer.

[31] Bhutani D.N., Goel D.V., Kajal D.P., Pawar D.D., Sharma D.P., Sen D.R. (2020). Primary extra nodal Non-Hodgkin’s lymphoma of urinary bladder presenting as a bladder tumor: A case report. Ann. Med. Surg..

[32] Sain B., Blake M., Goyal K., Kaur H., Robinson K. (2023). Epstein–Barr virus-positive primary diffuse large B-cell lymphoma of the urinary bladder: A case report. J. Surg. Case Rep..

[33] Zheng H., Du H., Liu J. (2024). Primary Bladder Non-Hodgkin Lymphoma: A Case Report. Cureus.

[34] Page M.J., McKenzie J.E., Bossuyt P.M., Boutron I., Hoffmann T.C., Mulrow C.D., Shamseer L., Tetzlaff J.M., Akl E.A., Brennan S.E. (2021). The PRISMA 2020 statement: An updated guideline for reporting systematic reviews. BMJ.

