# Supplementary material for: Primary Bladder Lymphoma with Extravesical Extension: A Case Report and Literature Review on Prognosis and Clinical Characteristics
Source: J Clin Med. 2024 Jul 25;13(15):4340. doi: 10.3390/jcm13154340 (PMC11313225; doi:10.3390/jcm13154340)
Supplement: Supplementary file 1 [file jcm-13-04340-s001.zip › Document S1 - Literature Review Method and Study Selection.pdf]

Literature Review Method and Study Selection

This scoping review was conducted following the PRISMA-ScR (Preferred Reporting Items for Systematic Reviews and Meta-Analyses extension for Scoping Reviews) 2020 guidelines [1], provided in Supplementary Table S2. The objective was to systematically identify and review all reported cases of Primary Bladder Lymphoma with Extravesical Extension. A comprehensive literature search was conducted in PubMed and Web of Science by two authors (H.S. and A.T.) from January 1, 1970, to June 1, 2024. Discrepancies were resolved by a third reviewer. Additionally, we manually screened the references or citations of each article. The search strategy was developed using a combination of Medical Subject Heading (MeSH) terms and free-text keywords related to Primary Bladder Lymphoma and Extravesical Extension. The search terms included: "Primary Bladder Lymphoma" [MeSH Terms], "Primary Bladder Lymphoma" [All Fields], "Extravesical Extension" [All Fields], and "Perivesical Extension" [All Fields]. The search query was constructed as follows: “Primary” AND "Bladder" AND "Lymphoma".

Inclusion and Exclusion Criteria

Studies were included if they met the following criteria: reported cases of Primary Bladder Lymphoma with Extravesical Extension, published in English, and included sufficient clinical details for analysis. Exclusion criteria were: studies not involving primary bladder lymphoma, studies without evidence of extravesical extension, and non-English publications.

Data Sources and Management

The search was conducted in PubMed and Web of Science, and all identified records were imported into EndNote for reference management. Duplicate records were removed. Titles and abstracts of the remaining articles were screened independently by two reviewers. Full-text articles were then assessed for eligibility.

Data Extraction

Data were extracted from each included study by two independent reviewers (H.S. and A.T.) using a standardized data extraction form. The following data were collected: author(s), year of publication, patient demographics (age, gender), clinical presentation, diagnostic methods, treatment modalities, and outcomes. Discrepancies between reviewers were resolved through discussion and consensus. If necessary, a third reviewer was consulted.

Data Synthesis

Extracted data were synthesized qualitatively. Key findings were summarized in tables and narrative form. The frequency and patterns of clinical characteristics, diagnostic methods, treatments, and outcomes were analyzed.

Quality Assessment

Although a scoping review does not typically include a formal quality assessment of the included studies, the relevance and completeness of each study's data were critically appraised.

Reporting

The review findings were reported according to the PRISMA-ScR checklist. A flow diagram (Figure 3) was included to illustrate the study selection process, and the key findings were summarized in both narrative and tabular formats.

1. Page, M.J.; McKenzie, J.E.; Bossuyt, P.M.; Boutron, I.; Hoffmann, T.C.; Mulrow, C.D.; Shamseer, L.; Tetzlaff, J.M.; Akl, E.A.; Brennan, S.E. The PRISMA 2020 statement: an updated guideline for reporting systematic reviews. *bmj* **2021**, *372*.
