# Supplementary material for: Primary Bladder Lymphoma with Extravesical Extension: A Case Report and Literature Review on Prognosis and Clinical Characteristics
Source: J Clin Med. 2024 Jul 25;13(15):4340. doi: 10.3390/jcm13154340 (PMC11313225; doi:10.3390/jcm13154340)
Supplement: Supplementary file 1 [file jcm-13-04340-s001.zip › Table S1 Characteristics of patients with primary bladder lymphoma with perivesical expansion.pdf]

Case no.	Age	Sex	Histology	Presentation	Renal impairment	VTE	Treatment	Outcome	Cause of death	Reference
1	50	F	MZL/MALT lymphoma	NA	NA	NA	S, R	Lost to follow-up	NA	[6]
2	64	F	MZL/MALT lymphoma	NA	NA	NA	S	Alive after 20y	Unknown cause	[6]
3	73	F	MZL/MALT lymphoma	NA	NA	NA	Fulgration, R	Alive after 12y	Unknown cause	[6]
4	79	F	MZL/MALT lymphoma	NA	NA	NA	R	Alive after 10y	Myocardial infraction	[6]
5	27	F	MZL/MALT lymphoma	NA	NA	NA	S, R	Alive after 2y	Fibrosarcoma	[6]
6	45	M	MZL/MALT lymphoma	NA	NA	NA	S, R	Alive after 40y	Unknown primary adenocarcinoma	[6]
7	54	F	Malignant	NA	No	No	C	CCR,	-	[12]

			non-Hodgkin lymphoma					>26y		
8	52	M	T-LBL	Suprapubic pain, Hematuria	No	No	C	NA	NA	[13]
9	66	F	MZL/MALT lymphoma	NA	NA	NA	NA	CCR, >1y	-	[5]
10	79	F	MZL/MALT lymphoma	Hematuria	NA	NA	NA	No follow up	NA	[5]
11	59	F	MZL/MALT lymphoma	NA	NA	NA	NA	CCR, >3y	-	[5]
12	84	F	DLBCL	Hematuria	No	No	None	PD, died 6m	Lymphoma	[5]
13	67	M	DLBCL	Hematuria, pain	No	No	C, R	CCR, >16y	-	[5]
14	80	F	DLBCL	Hematuria	Yes	Yes	R	CCR, >4y	-	[5]
15	75	F	MZL/MALT	Hematuria	No	No	C, R	CCR, >3y	-	[14]

			lymphoma							
16	40	M	DLBCL	Hematuria	No	No	C, R	CCR, >2y	-	[15]
17	30	F	PTCL	Hematuria	No	No	None	Died 0.5m	Lymphoma	[16]
18	35	M	DLBCL	Hematuria, flank pain, hydronephrosis	Yes	Yes	C	CCR, >18m	-	[17]
19	89	F	DLBCL	Urinary obstruction	No	No	C	Died 1y	Lymphoma	[18]
20	69	F	DLBCL	NA	No	No	S, C	PD	Lymphoma	[19]
21	82	F	MZL/MALT lymphoma	Hematuria	NA	NA	S, C	Died	NA	[7]
22	87	F	B-cell	Recurrent urinary tract infection	NA	NA	Antibiotics	Alive after 6y	-	[7]
23	75	F	B-cell	Hematuria	NA	NA	C	Died	Lymphoma	[7]
24	81	F	MZL/MALT lymphoma	Hematuria	NA	NA	Diathermy	Alive after 1y	-	[7]
25	27	M	Anaplastic large cell	Hemturia, left iliac fossa pain	NA	NA	S, C	Alive after 7y	-	[7]

			lymphoma							
26	28	M	MZL/MALT lymphoma	Hematuria	NA	NA	SC	Alive after 10y	-	[7]
27	76	F	MZL/MALT lymphoma	Hematuria	NA	NA	R	Alive after 2y	-	[7]
28	77	M	MZL/MALT lymphoma	Hematuria	NA	NA	C	Alive after 4y	-	[7]
29	31	M	DLBCL	Recurrent urinary tract infection	NA	NA	S, C	Alive after 8y	-	[7]
30	70	F	DLBCL	Hematuria	NA	NA	S, C	Alive after 4y	-	[7]
31	66	F	MZL/MALT lymphoma	Recurrent urinary tract infection	NA	NA	R	Died	NA	[7]
32	74	F	MZL/MALT lymphoma	Recurrent urinary tract infection	No	No	C	CCR, >4y	-	[20]
33	64	F	DLBCL	Hematuria, pollakisuria,	No	No	C	SD	NA	[21]

				urinary incontinence, hydronephrosis						
34	54	M	B-cell	Dysuria, night sweting	No	No	C	CR, relapsed in the kidney after 9y	NA	[22]
35	65	F	B-cell	Dysuria, urinary urgency, Hematuria, pollakisuria	No	No	S, C, R	CCR, >1y	-	[23]
36	75	F	DLBCL	Oliguria, hydrpnephrosis	Yes	Yes	C	NA	NA	[24]
37	79	M	DLBCL	Hematuria	No	No	C	CCR	-	[25]
38	45	M	PTCL	Hematuria, dysuria, loin pain	No	No	C	CCR, >12m	-	[26]
39	69	M	DLBCL	Hematuria, dysuria	No	No	C	CCR, >6m	-	[27]
40	57	F	DLBCL	Bladder irritation, dysuria,	NA	NA	C	CCR, >4y	-	[28]

				urinary urgency, hematuria						
41	48	M	DLBCL	Hematuria, nocturia, flank pain, pollakisuria, hydroureteronephrosis	NA	NA	C	CCR	-	[29]
42	66	M	DLBCL	NA	NA	NA	S, C	NA	NA	[30]
43	40	M	DLBCL	Hematuria	No	No	C, R	CCR, >2y	-	[31]
44	80	F	DLBCL	Abdominalgia, fever, constipation, vomining, back pain, pollakisuria	Yes	Yes	S	Died	Lymphoma	[32]
45	83	F	DLBCL	Polyuria	No	No	C	CCR	NA	[33]
46	50	M	PTCL	Asymptomatic	No	No	S, C	NA	NA	[33]
47	77	M	DLBCL	UTI, anorexia, malise, renal failure	Yes	Yes	C	PD, after cycle 3	-	Present case
